# Accuracy of Dengue Reporting by National Surveillance System, Brazil

**DOI:** 10.3201/eid2202.150495

**Published:** 2016-02

**Authors:** Monaise M.O. Silva, Moreno S. Rodrigues, Igor A.D. Paploski, Mariana Kikuti, Amelia M. Kasper, Jaqueline S. Cruz, Tássia L. Queiroz, Aline S. Tavares, Perla M. Santana, Josélio M.G. Araújo, Albert I. Ko, Mitermayer Galvão Reis, Guilherme S. Ribeiro

**Affiliations:** Centro de Pesquisas Gonçalo Moniz, Fundação Oswaldo Cruz, Salvador, Brazil (M.M.O. Silva, M.S. Rodrigues, I.A.D. Paploski, M. Kikuti, A.M. Kasper, J.S. Cruz, T.L. Queiroz, A.S. Tavares, P.M. Santana, A.I. Ko, M.G. Reis, G.S. Ribeiro);; nstituto de Saúde Coletiva, Universidade Federal da Bahia, Salvador (M.M.O Silva, I.A.D. Paploski, M. Kikuti, T.L. Queiroz, G.S. Ribeiro);; Universidade Federal do Rio Grande do Norte, Natal, Brazil (J.M.G. Araújo);; Yale University School of Public Health, New Haven, Connecticut, USA (A.I. Ko, M.G. Reis, G.S. Ribeiro);; Faculdade de Medicina, Universidade Federal da Bahia, Salvador (M.G. Reis)

**Keywords:** dengue, acute febrile illness, dimensional measurement accuracy, disease notification, mandatory reporting, infectious disease reporting, viruses, Brazil, Sistema de Informação de Agravos de Notificação, SINAN

**To the Editor:** Dengue is an underreported disease globally. In 2010, the World Health Organization recorded 2.2 million dengue cases (*1*), but models projected that the number of symptomatic dengue cases might have been as high as 96 million (*2*). Brazil reports more cases of dengue than any other country (*1*); however, the degree of dengue underreporting in Brazil is unknown. We conducted a study to evaluate dengue underreporting by Brazil’s Notifiable Diseases Information System (Sistema de Informação de Agravos de Notificação [SINAN]).

From January 1, 2009, through December 31, 2011, we performed enhanced surveillance for acute febrile illness (AFI) in a public emergency unit in Salvador, Brazil. The surveillance team enrolled outpatients >5 years of age with measured (>37.8°C) or reported fever. Patients or their legal guardians provided written consent. The study was approved by the Oswaldo Cruz Foundation Ethics Committee, Brazil’s National Council for Ethics in Research, and the Yale Institutional Review Board.

We collected participants’ blood samples at study enrollment and >15 days later. Acute-phase serum samples were tested by dengue nonstructural protein 1 ELISA and IgM ELISA (Panbio Diagnostics, East Brisbane, Queensland, Australia). Convalescent-phase serum samples were tested by IgM ELISA. In concordance with case-reporting guidelines in Brazil (*3*), we defined dengue cases by a positive nonstructural protein 1 ELISA result or a positive acute-phase or convalescent-phase IgM ELISA result. All others were classified as nondengue AFI.

We then identified which study patients were officially reported to SINAN as having a suspected case of dengue. In Brazil, notification of suspected dengue cases is mandatory. A suspected case is defined as illness in a person from an area of dengue transmission or *Aedes aegypti* mosquito infestation who has symptoms of dengue (fever of <7 days’ duration, plus >2 of the following symptoms: nausea/vomiting, exanthema, myalgia, arthralgia, headache, retro-orbital pain, petechiae/positive tourniquet test, or leukopenia). We used Link Plus software (CDC-Link Plus Production 2.0; Centers for Disease Control and Prevention, Atlanta, GA, USA) to perform probabilistic record linkage from our database with official reports in the SINAN database. The records were matched based on the patients’ first names, last names, and dates of birth. We then manually reviewed the matches to confirm the pairs.

On the basis of the results, we calculated the sensitivity, specificity, positive predictive value (PPV), and negative predictive value of the national surveillance system. We calculated accuracy measurements with 95% CIs for the overall study period and for each study year, age group (5–14 vs. >15 years), and seasonal prevalence of dengue (months of low vs. high dengue transmission, defined by dengue detection in <20% vs. >20% of the AFI patients, respectively). We estimated multiplication factors by dividing the number of dengue cases in our study by the number of study patients who were reported to SINAN as having dengue. 

Of the 3,864 AFI patients identified during the 3-year study period, 997 (25.8%) had laboratory evidence of dengue infection, and 2,867 (74.2%) were classified as having nondengue AFI. Of the 997 dengue cases, 57 were reported to SINAN (sensitivity 5.7%) (Table). Of the 2,867 nondengue AFI cases, 26 were reported to SINAN as dengue cases (false-positive ratio 0.9%, specificity 99.1%). None of these 26 cases had laboratory confirmation in the SINAN database. The PPV for reporting to SINAN was 68.7%, and the negative predictive value was 75.1% (Table). PPV was higher among patients >15 years of age, which might be attributable to atypical presentations of dengue in children (*4*,*5*).

**Table:** Accuracy of a national surveillance system for recording cases of suspected dengue among patients with acute febrile illness who visited an emergency health unit of Salvador, Brazil, January 1, 2009–December 31, 2011*

Notification status of AFI patients†	Laboratory status of AFI patients, no.‡		Dengue prevalence, %§	% (95% CI)				MF¶
	Dengue	Nondengue AFI		SENS	SPEC	PPV	NPV	
Overall								
Reported	57	26	25.8	5.7 (4.4–7.3)	99.1 (98.7–99.4)	68.7 (58.1–77.6)	75.1 (73.7–76.5)	12.0
Not reported	940	2,841						
Study year								
2009								
Reported	4	4	10.3	3.3 (1.3–8.3)	99.6 (99.0–99.9)	50.0 (21.5–78.5)	90.0 (88.1- 91.6)	15.0
Not reported	116	1,039						
2010								
Reported	27	8	33.8	6.0 (4.1–8.6)	99.1 (98.2–99.5)	77.1 (61.0–87.9)	67.4 (64.8- 69.9)	12.9
Not reported	423	873						
2011								
Reported	26	14	31.2	6.1 (4.2–8.8)	98.5 (97.5–99.1)	65.0 (49.5–77.9)	69.9 (67.3- 72.3)	10.7
Not reported	401	929						
Age group, y								
5–14								
Reported	23	15	25.5	5.8 (3.9–8.6)	98.7 (97.9–99.2)	60.5 (44.7–74.4)	75.4 (73.2–77.5)	10.4
Not reported	372	1,139						
≥15								
Reported	34	11	26.0	5.6 (4.0–7.8)	99.4 (98.9–99.6)	75.6 (61.3–85.8)	75.0 (73.2–76.7)	13.8
Not reported	568	1,702						
Monthly dengue prevalence§								
≥20%								
Reported	52	18	38.1	6.7 (5.2–8.7)	98.6 (97.7–99.1)	74.3 (63.0–83.1)	63.1 (61.0–65.2)	11.1
Not reported	722	1,235						
≥20%								
Reported	5	8	12.1	2.2 (1.0–5.2)	99.5 (99.0–99.8)	38.5 (17.7–64.5)	88.0 (86.5–89.5)	17.2
Not reported	218	1,606						


*AFI, acute febrile illness; SINAN, Sistema de Informação de Agravos de Notificação (Notifiable Diseases Information System, Brazil); SENS, sensitivity; SPEC, specificity; PPV, positive predictive value; NPV, negative predictive value; MF, multiplication factor. †Notification status of the AFI patients was ascertained from the SINAN database on reported dengue cases for the city of Salvador. SINAN database was obtained from the Salvador Secretary of Health in January 2013. ‡Acute-phase serum samples from AFI patients were systematically tested for dengue by nonstructural protein 1 ELISA and IgM ELISA; convalescent-phase serum samples were also tested by IgM ELISA. §Among AFI patients assisted at the sentinel surveillance emergency unit. ¶Laboratory-confirmed dengue/AFI patients reported as having a suspected case of dengue.

We found that 1 in 4 patients with AFI had laboratory evidence of dengue infection. However, for every 20 dengue patients that we identified, only about 1 had been reported to SINAN as having dengue. During periods of low dengue transmission, only about 1 in 40 dengue cases identified was reported. Conversely, among the patients who were reported as having dengue, 31.2% did not have the disease; this percentage reached 61.5% in low-transmission periods.

We estimated that overall, there were 12 dengue cases per reported case in the community, but in months of low dengue transmission, this ratio was >17:1 (Table). Comparable results have been observed in Nicaragua, Thailand, and Cambodia (*6*–*8*). By applying the estimated multiplication factor to the study period’s mean annual incidence of 303.8 reported dengue cases/100,000 Salvador residents (*9*), we estimated that the actual mean annual dengue incidence for Salvador was 3,645.7 cases/100,000 residents.

We showed that dengue surveillance substantially underestimated disease burden in Brazil, especially in what are considered low-transmission periods. Dengue underreporting has been attributed to passive case detection, which fails to identify persons with dengue who do not seek health care (*1*). We also showed that surveillance failed to detect dengue cases among symptomatic patients seeking health care.

Novel surveillance tools, such as active syndromic surveillance and point-of-care testing, should be applied to improve estimates of dengue incidence. Furthermore, given the recent emergence of chikungunya and Zika viruses in Brazil (*10*), improved surveillance and laboratory diagnostics are needed to avert misclassification and mismanagement of cases.
